# Supplementary material for: Boosting spontaneous orientation polarization of polar molecules based on fluoroalkyl and phthalimide units
Source: Nat Commun. 2024 Oct 29;15:9297. doi: 10.1038/s41467-024-53633-3 (PMC11522372; doi:10.1038/s41467-024-53633-3)
Supplement: Supplementary file 1 — Supplementary Information [file 41467_2024_53633_MOESM1_ESM.pdf]

Supplementary Information

Boosting spontaneous orientation polarization of polar molecules based on fluoroalkyl and phthalimide units

Masaki Tanaka*[1]

1. Department of Biotechnology and Life Science, Tokyo University of Agriculture and Technology, 2-24-16 Naka-cho, Koganei, Tokyo 184-8588, Japan

E-mail: m-tanaka@me.tuat.ac.jp

Supplementary information

Supplementary Fig. 1. 6FDI-2*p*BNMe 4
Supplementary Fig. 2. DSC results 5
Supplementary Fig. 3. T_s dependence 6
Supplementary Fig. 4. PDM distribution 7
Supplementary Table 2. Summary of film properties 8
Supplementary Fig. 5. Film absorption 9
Supplementary Fig. 6. Vibration-based generated current 10
Supplementary Table 3. HOMO and LUMO energy levels. 11
Supplementary Fig. 7. PYS results 12
Supplementary Fig. 8. Photoluminescence spectra 13
Supplementary Fig. 9. Carrier transport properties 14
Supplementary Table 4. Charge mobility 15
Supplementary Fig. 10. Computational calculated energy levels of 6FDI-2*p*BN 16
Supplementary Fig. 11. Computational calculated energy levels of 6FDI-2*o*BN 17
Supplementary Fig. 12. OLEDs with SOP-ETLs 18
Supplementary Fig. 13. PL and EL lifetimes 19
Supplementary Fig. 14. DCM profiles of OLEDs with SOP-ETLs 20
Supplementary Fig. 15. SOP-interlayers 21
Supplementary Fig. 16. DCM profiles of OLEDs with SOP-interlayers 22
Supplementary Table 5. OLED performance 23
Supplementary Figs. 17-30. NMR results 24
Supplementary References 31

Supplementary Table 1. List of GSP slope values

Supplementary Table 1. List of GSP slope values reported in literature.

Polar molecule	PDM (Debye)	GSP slope (mV/nm)
a-NPD[1]	0.34	+5.3
TPBi[1]	2.0	+43
BAlq[1]	2.32	+25
OXD-7[1]	3.77	+68
Alq_3[1]	4.4	+48
$Ir(ppy)_3$[1]	6.41	−3.6
mCP[1]	1.35	−3.9
BCP[1]	2.9	+33
$Al(7\text{-}prq)_3$[1]	3.76	−103
$Al(q\text{-}Cl)_3$[1]	3.81	+94
Gaq_3[1]	4.45	+47
Znq_2[1]	5.13	+5.8
4CzPN[1]	6.49	+40
DACT-II[1]	2.02	+13
$Ir(ppy)_2(acac)$[1]	2.53	+38
4CzIPN[1]	3.85	+51
Bpy-OXD[1]	3.86	+37
B3PyMPM[1]	4.29	+3
2CzPN[1]	7.04	+58
DCJTB[1]	15.5	+14.8
BCPO[2]	3.5	+151
p-ethyl-TPBi[3]	7.0	+141
6F-2TRZ[4]	2.97	-108
6F-2Cz[4]	0.98	−40
6F-TPA-TRZ[4]	3.19	−46
6F-Cz-TRZ[4]	2.53	−63
6F-2BN[4]	3.16	+69
3F-3BN[4]	3.02	+130
6F-Cz-TRZ[4]	2.53	−61

Supplementary Fig. 1. 6FDI-2*p*BNMe

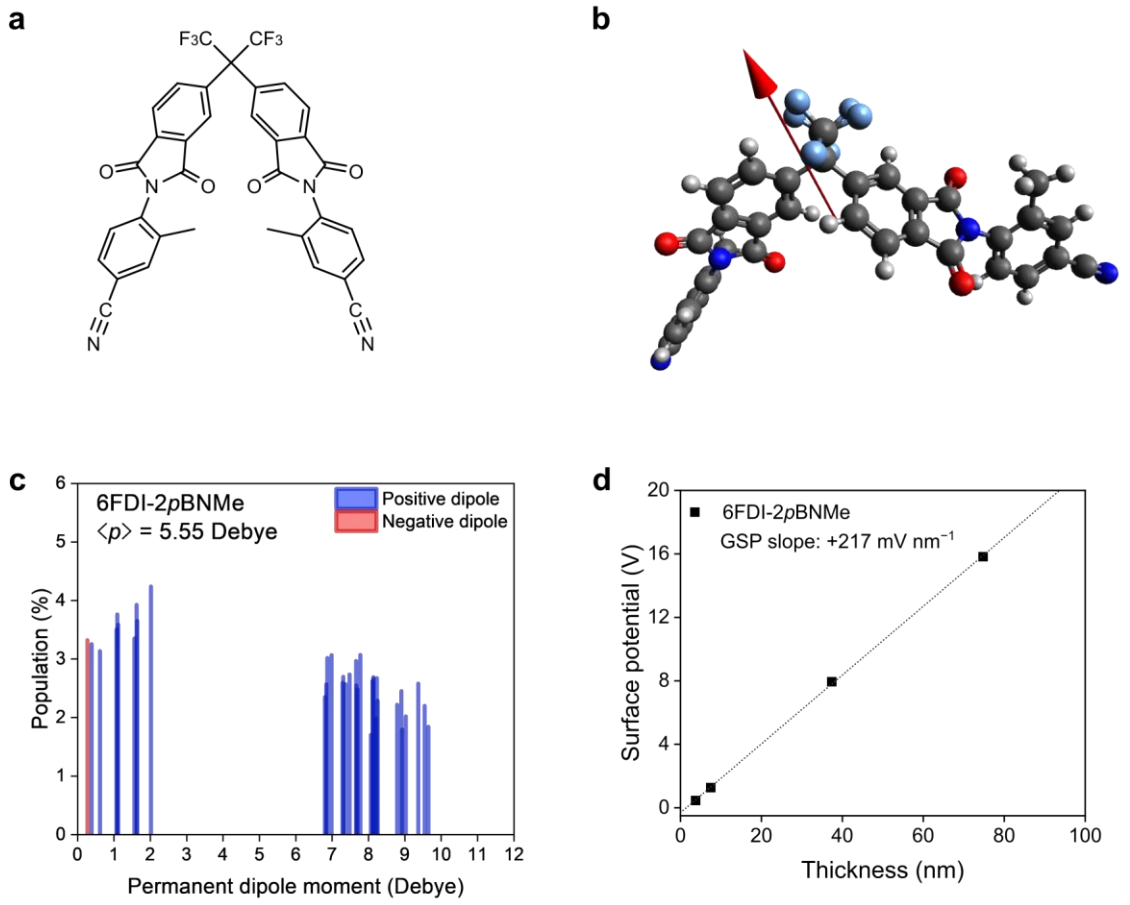

Supplementary Fig. 1. 6FDI-2*p*BNMe. **a,** Molecular structure of 6FDI-2*p*BNMe. **b,** Calculated PDM direction. **c,** Distribution of PDM magnitudes of the conformers. **d,** Thickness dependence of surface potentials of a 6FDI-2*p*BNMe film.

Supplementary Fig. 2. DSC results

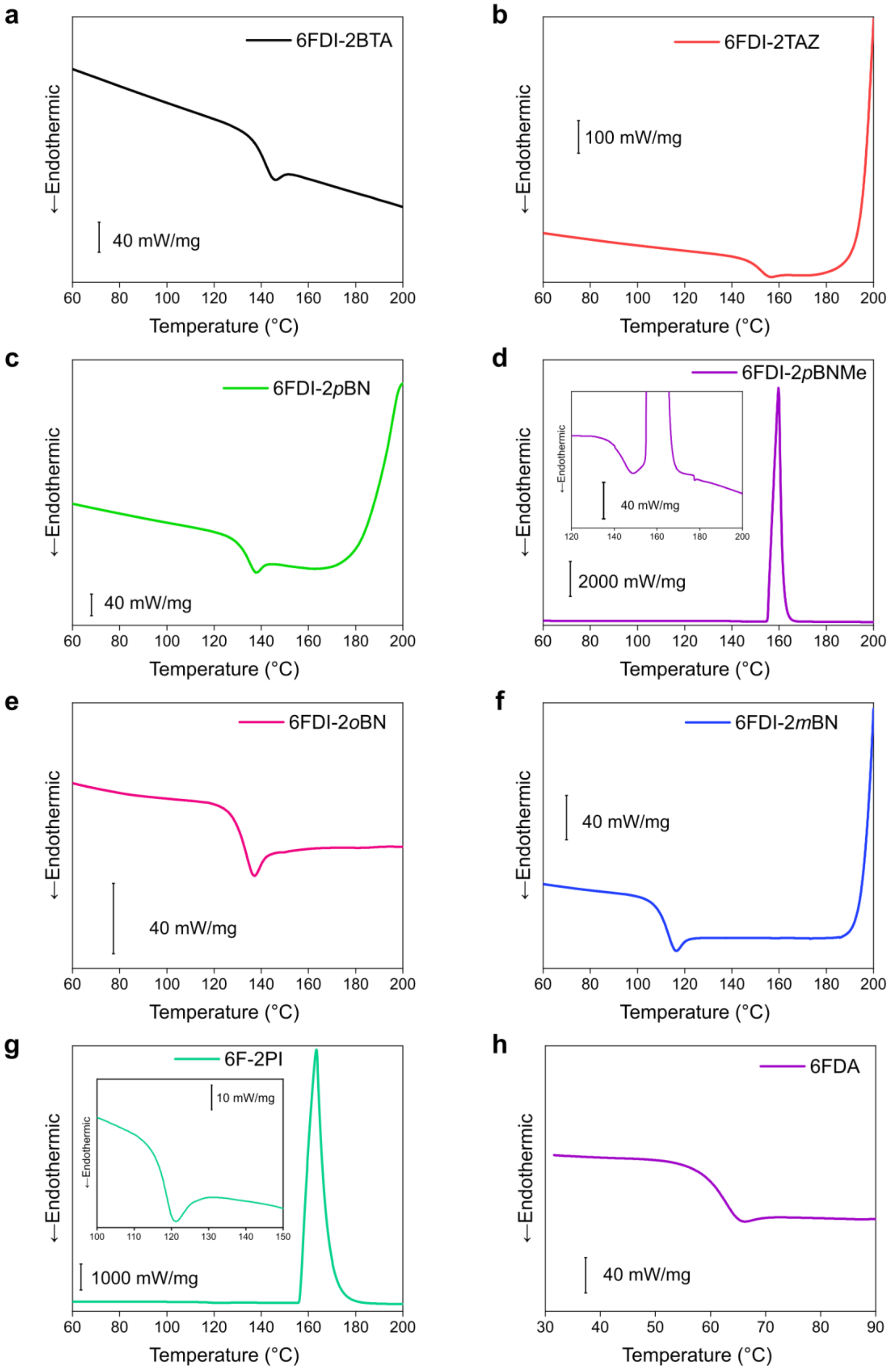

Supplementary Fig. 2. Differential scanning calorimetry (DSC) measurement results. **a,** 6FDI-2BTA. **b,** 6FDI-2TAZ. **c,** 6FDI-2*p*BN. **d,** 6FDI-2*p*BNMe. **e,** 6FDI-2*o*BN. **f,** 6FDI-2*m*BN. **g,** 6F-2PI. **h,** 6FDA.

Supplementary Fig. 3. T_s dependence

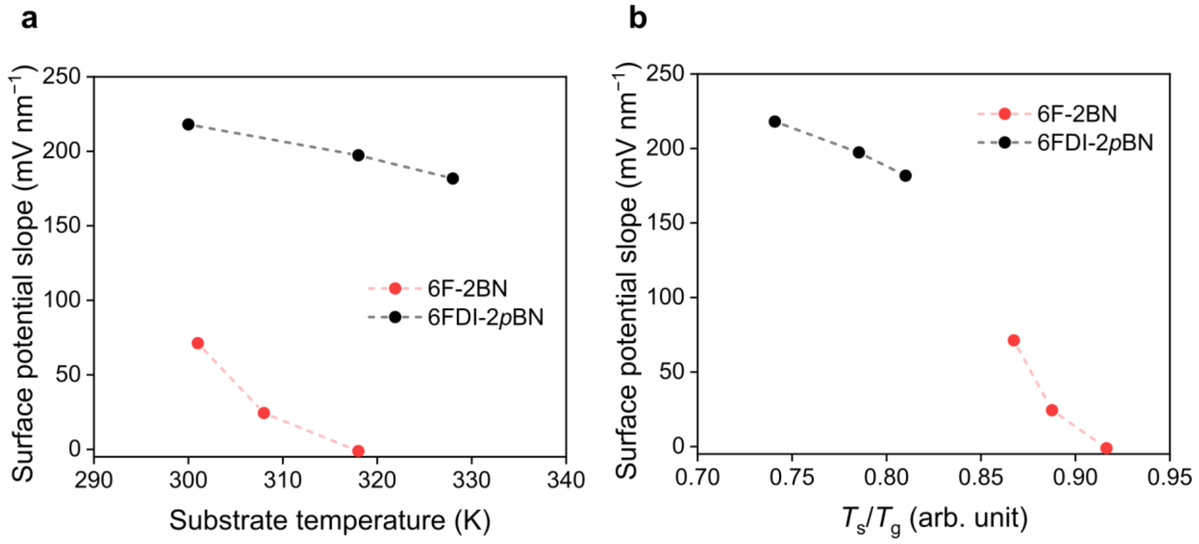

Supplementary Fig. 3. T_s dependence of surface potential slope. **a,** Substrate temperature (T_s) dependence of the surface potential slopes of 6F-2BN and 6FDI-2*p*BN. **b,** T_s/glass transition temperature (T_g) dependence of surface potential slopes.

Supplementary Fig. 4. PDM distribution

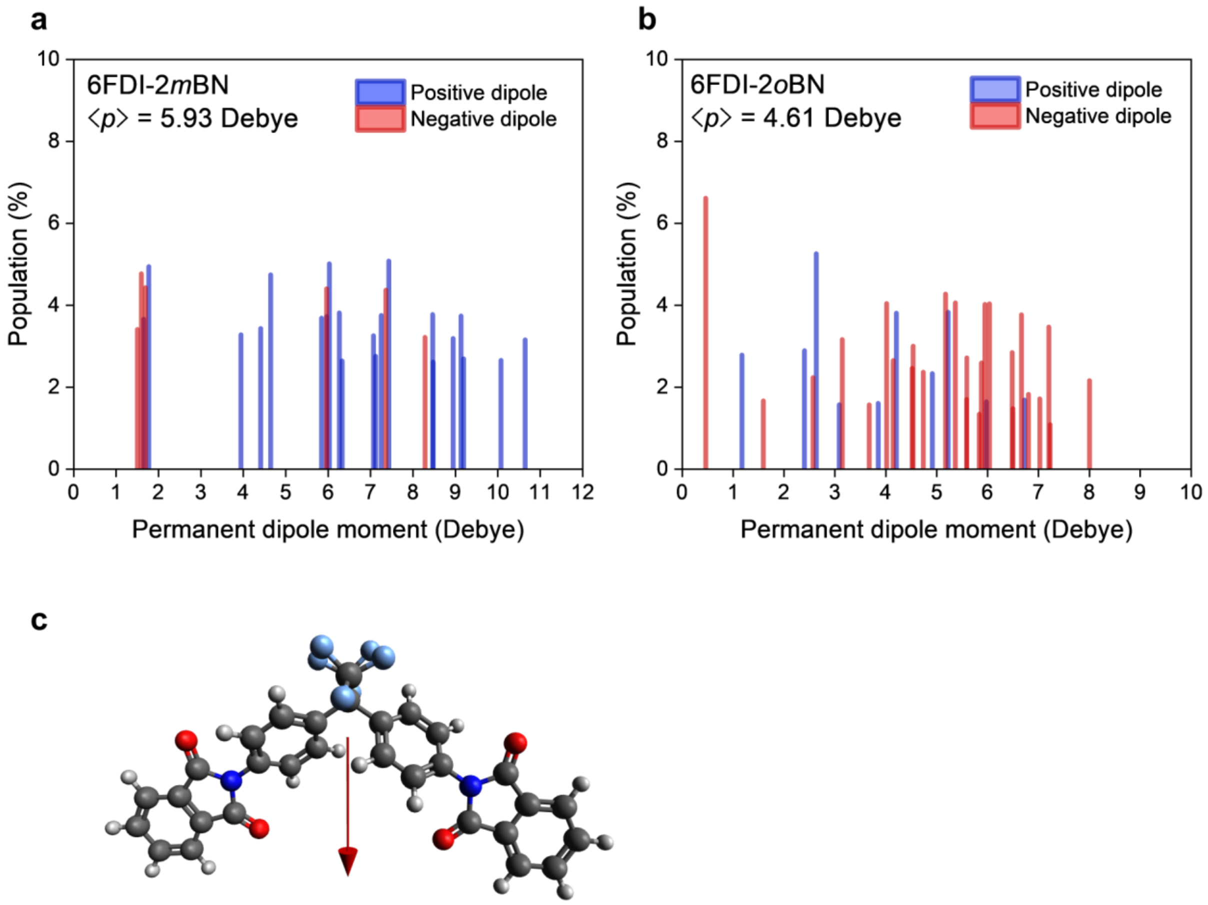

Supplementary Fig. 4. Distributions of permanent dipole moment magnitude. **a,** 6FDI-2*m*BN. **b,** 6FDI-2*o*BN. **c,** Calculated PDM of 6F-2PI (5.82 Debye).

Supplementary Table 2. Summary of film properties

Supplementary Table 2. Summary of properties of polar films deposited at a deposition rate of ~0.1 nm s^{-1}.

	T_g [a] (°C)	$\langle p \rangle$ [b] (Debye)	GSP slope (mV nm^{-1})	σ [c] (mC m^{-2})	$\langle \cos\theta \rangle$ [d] (arb. unit)
6FDI-2*p*BNMe	138	5.55	+217	+5.75	+0.18
6FDI-2*o*BN	130	4.61	−103	−2.73	−0.10
6FDI-2*m*BN	111	5.93	+87	+2.31	+0.07
6F-2PI	119	5.82	−210	−5.56	−0.17

[a]Glass transition temperature. [b]Average permanent dipole moment magnitude. [c]Surface charge density. [d]Average orientation degree.

Supplementary Fig. 5. Film absorption

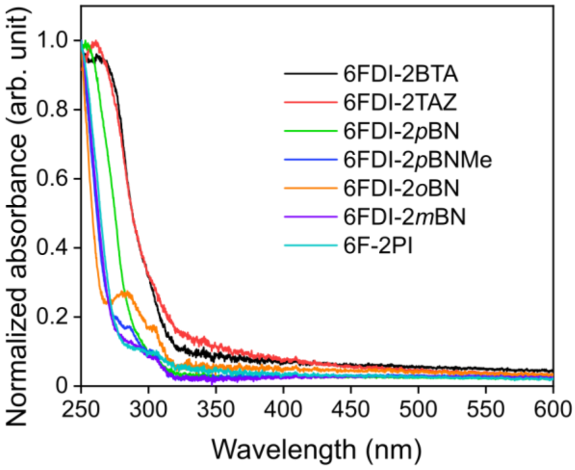

Supplementary Fig. 5. Absorption spectra of vacuum-deposited films of developed polar molecules.

Supplementary Fig. 6. Vibration-based generated current

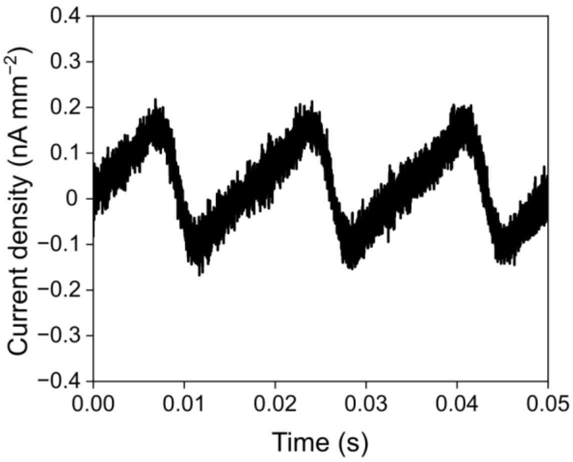

Supplementary Fig. 6. Vibration-based generated current profile of the vacuum-deposited electret film. The probe vibrated above the 6FDI-2*p*BN film (surface potential~20 V), and the generated current was collected using an oscilloscope and a current/voltage amplifier. The current values were normalized by the probe area (12.57 mm^2) because the area of the deposited film (25×25 mm^2) was larger than the probe area.

Supplementary Table 3. HOMO and LUMO energy levels.

Supplementary Table 3. Summary of E_{HOMO}, E_{LUMO}, and E_{gap}.

	E_{HOMO} (eV)[a]	E_{LUMO} (eV)[b]	E_{gap} (eV)[c]
6FDI-2BTA	7.11	3.29	3.82
6FDI-2TAZ	7.14	3.27	3.87
6FDI-2*p*BN	7.76	3.92	3.84
6FDI-2*m*BN	7.72	3.89	3.83
6FDI-2*o*BN	7.87	3.98	3.89
6F-2PI	7.56	3.80	3.76
6FDI-2*p*BNMe	7.72	3.81	3.91

[a]Energy level of HOMO. [b]Energy level of LUMO. [c]Estimated from optical bandgap energy.

Supplementary Fig. 7. PYS results

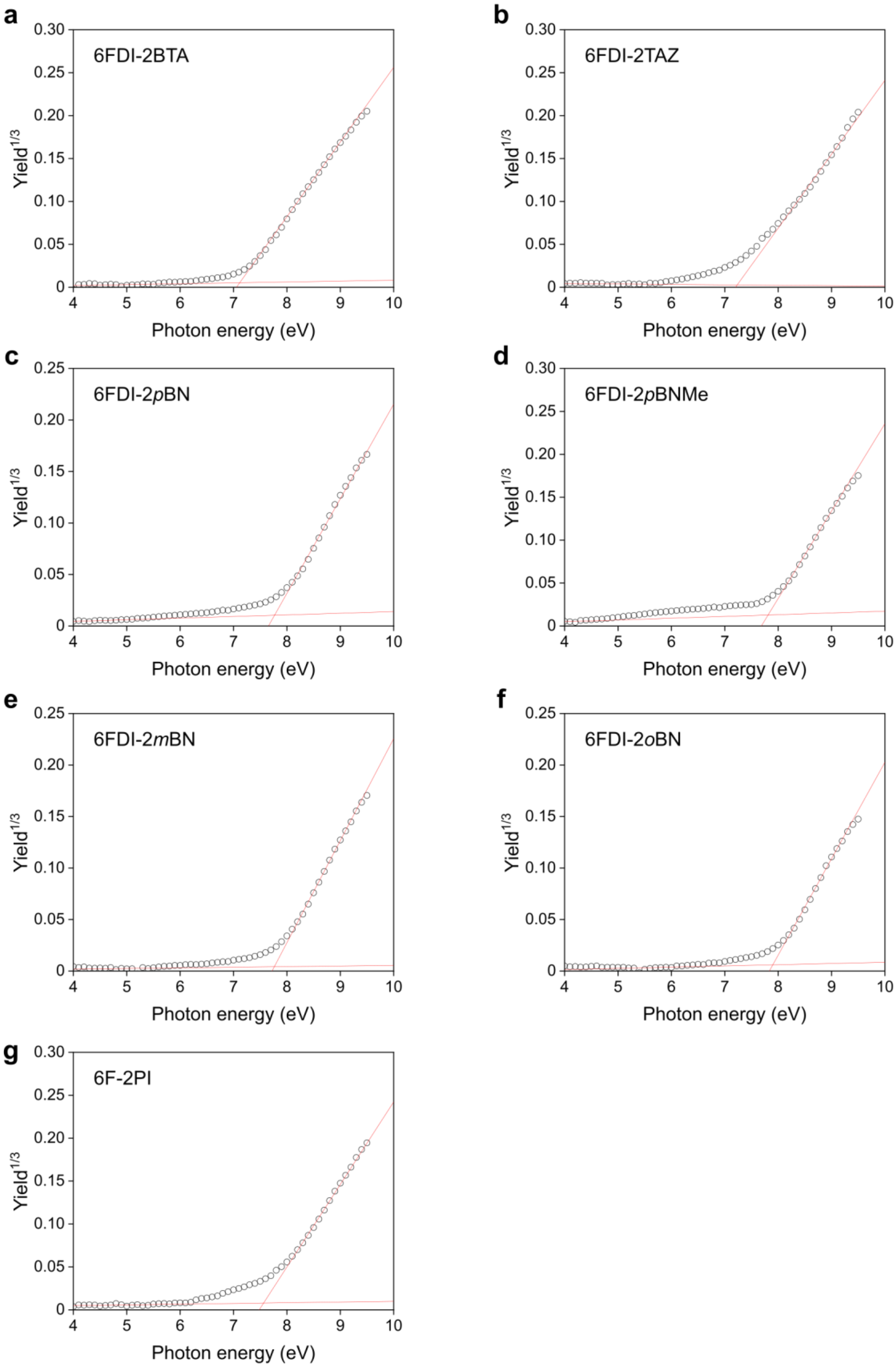

Supplementary Fig. 7. Photoelectron yield spectroscopy results. **a,** 6FDI-2BTA. **b,** 6FDI-2TAZ. **c,** 6FDI-2*p*BN. **d,** 6FDI-2*p*BNMe. **e,** 6FDI-2*m*BN. **f,** 6FDI-2*o*BN. **g,** 6F-2PI.

Supplementary Fig. 8. Photoluminescence spectra

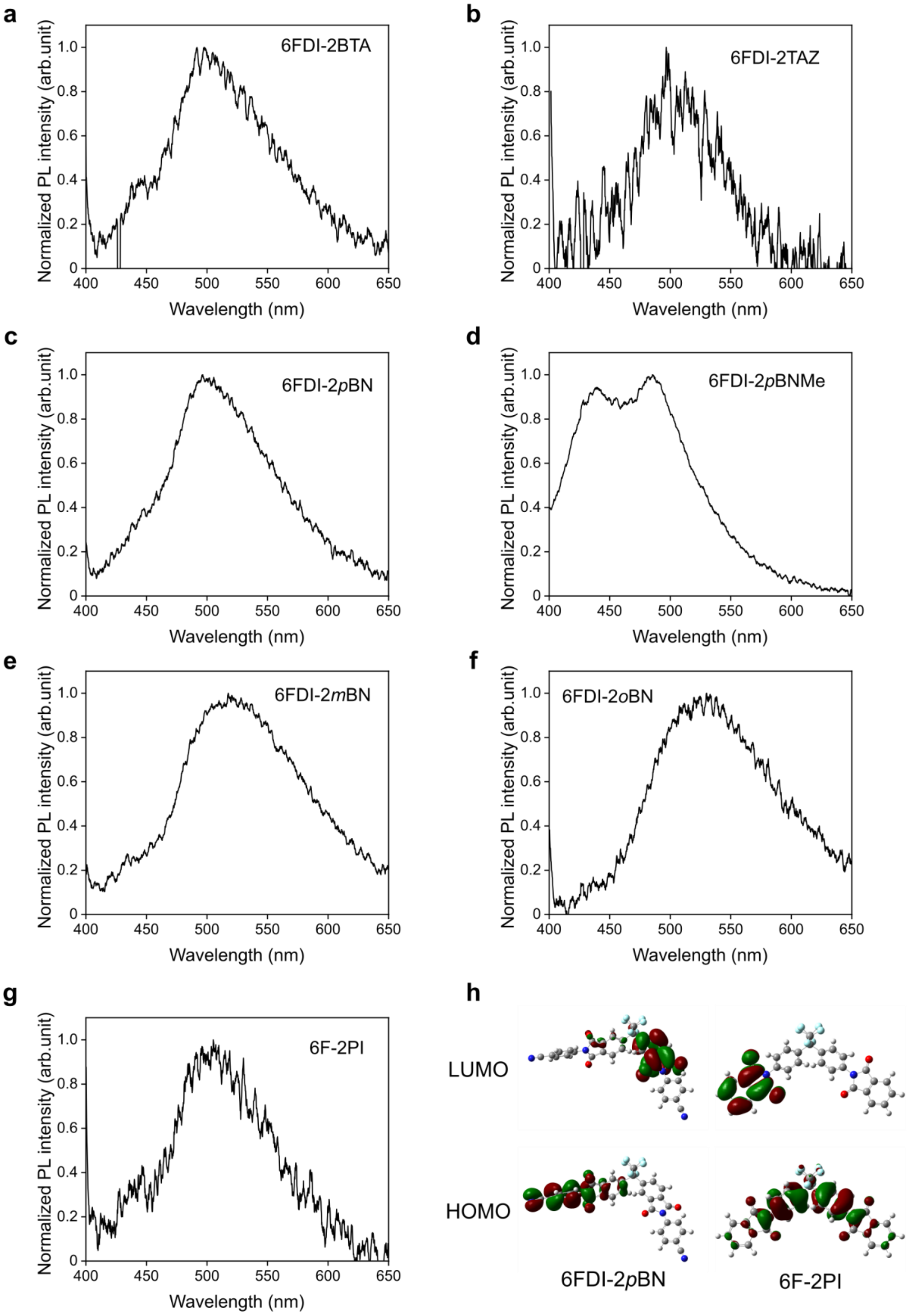

Supplementary Fig. 8. Photoluminescence (PL) spectra. **a,** 6FDI-2BTA. **b,** 6FDI-2TAZ. **c,** 6FDI-2*p*BN. **d,** 6FDI-2*p*BNMe. **e,** 6FDI-2*m*BN. **f,** 6FDI-2*o*BN. **g,** 6F-2PI. Vacuum-deposited films were excited using 365 nm-LED. **h,** Calculated HOMO and LUMO distributions of 6FDI-2*p*BN and 6F-2PI.

Supplementary Fig. 9. Carrier transport properties

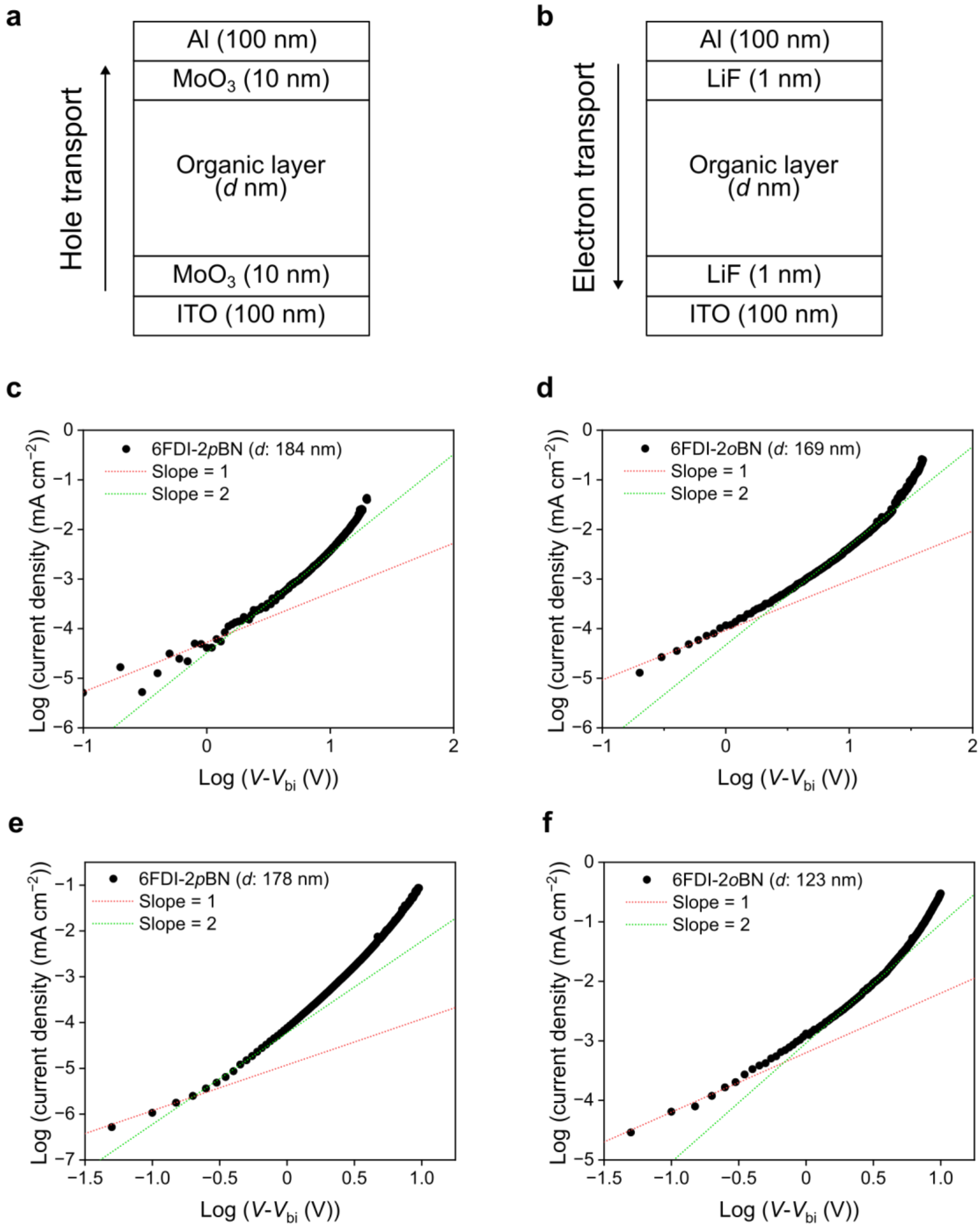

Supplementary Fig. 9. Carrier transport properties. **a,** Hole-only device (HOD) structure. **b,** Electron-only device (EOD) structure. **c** and **d,** Current density-voltage (*V*) characteristics of HODs based on 6FDI-2*p*BN (c) and 6FDI-2*o*BN (d). **e** and **f,** Current density-V characteristics of EODs based on 6FDI-2*p*BN (e) and 6FDI-2*o*BN (f). V_{bi} represents built-in potential.

Supplementary Table 4. Charge mobility

Supplementary Table 4. Summary of zero-field hole and electron mobilities (μ_{0h} and μ_{0e}) estimated using the Child's law.

	μ_{0h} (cm^2 V^{-1} s^{-1})[a]	μ_{0e} (cm^2 V^{-1} s^{-1})[b]
6FDI-2*p*BN	6.8×10^{-10}	1.1×10^{-9}
6FDI-2*o*BN	7.6×10^{-10}	5.7×10^{-9}

[A]Zeho-field hole mobility. [b]Zero-field electron mobility.

Supplementary Fig. 10. Computational calculated energy levels of 6FDI-2*p*BN

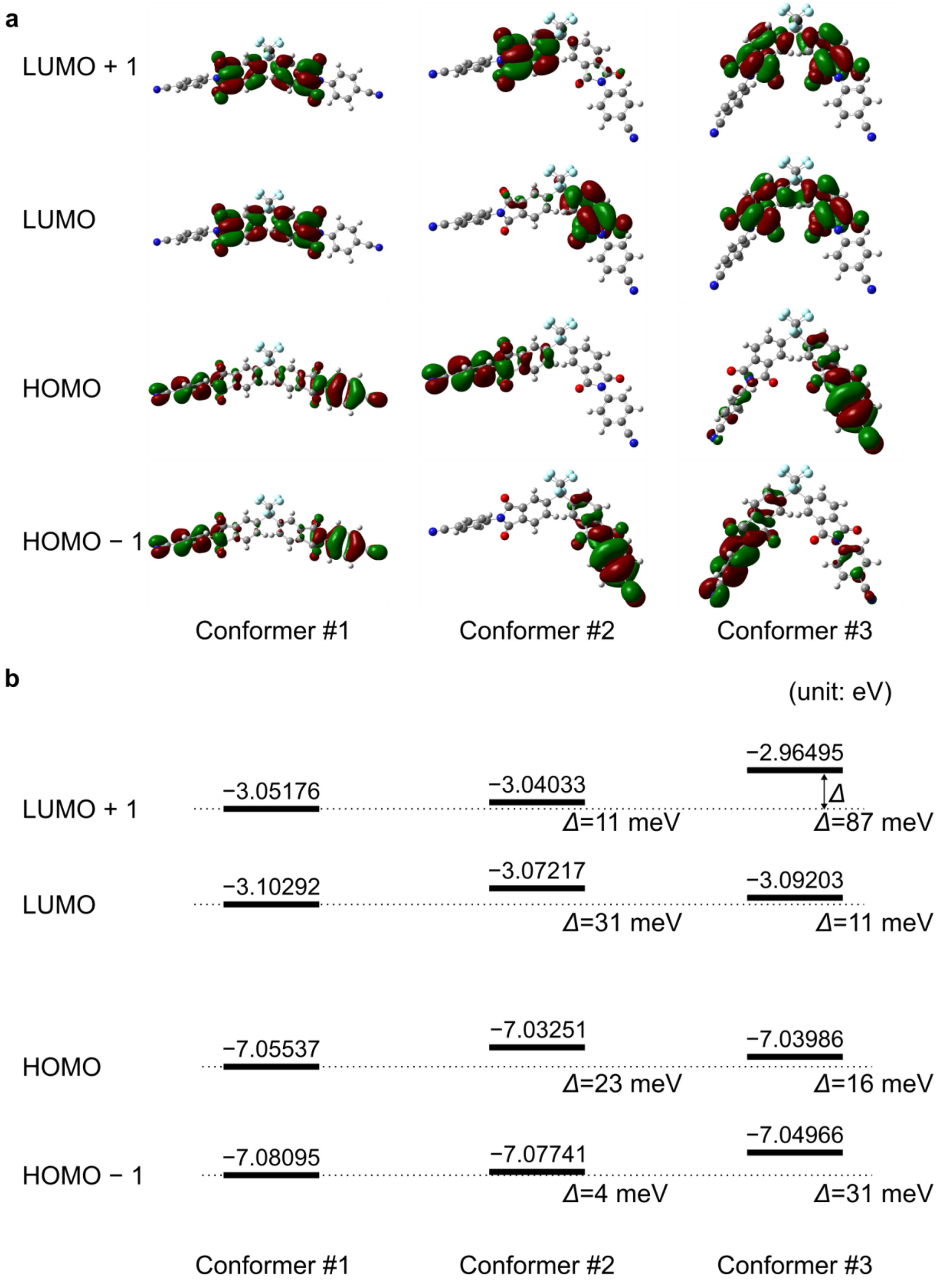

Supplementary Fig. 10. Computational calculated highest occupied molecular orbital (HOMO) and lowest unoccupied molecular orbital (LUMO) levels of 6FDI-2*p*BN. **a,** HOMO and LUMO distributions. **b,** Energy alignment of LUMO+1, LUMO, HOMO, and HOMO−1. The *Δ* values denote the energy level differences between the conformer #1 and each conformer.

Supplementary Fig. 11. Computational calculated energy levels of 6FDI-2*o*BN

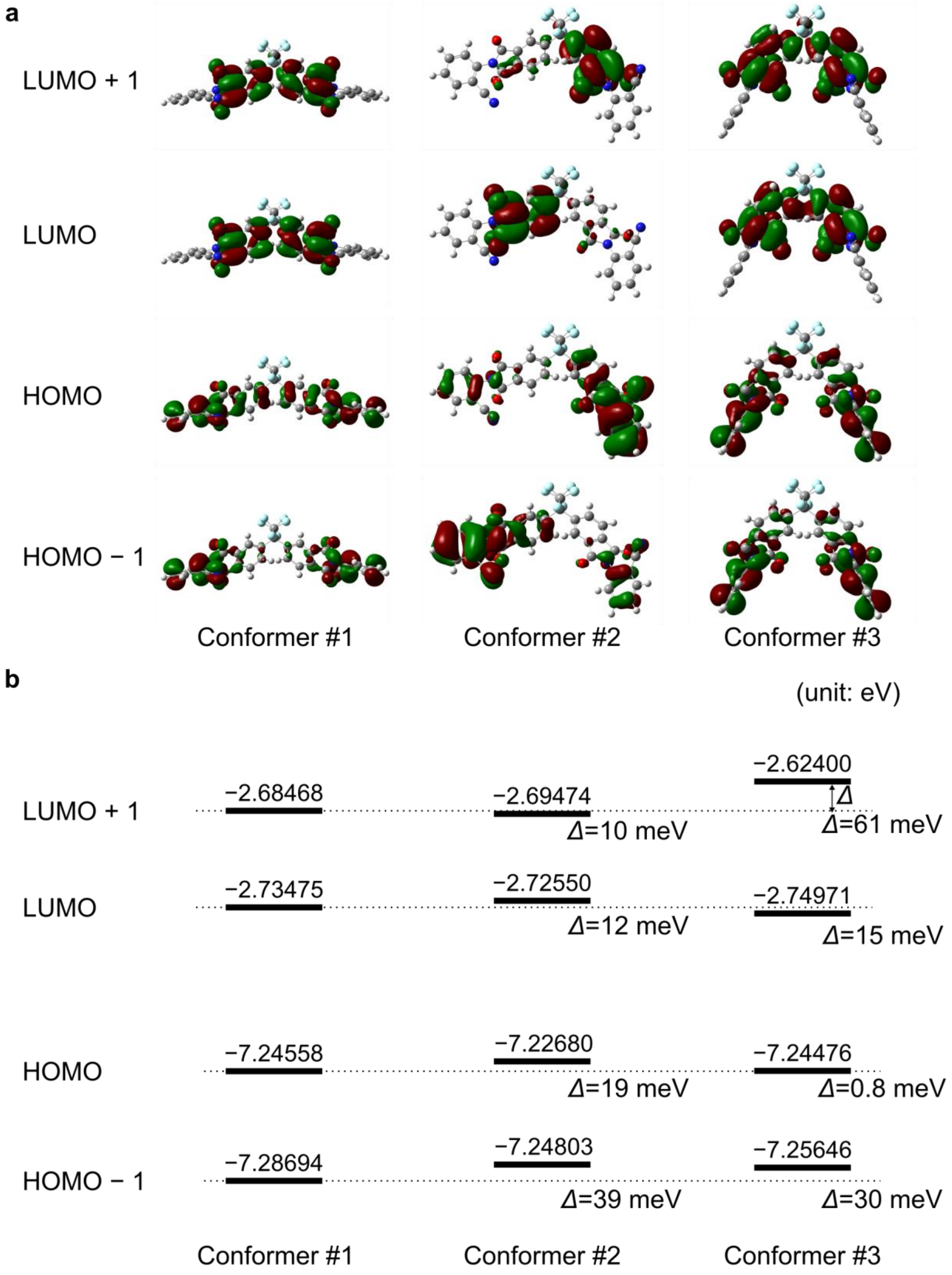

Supplementary Fig. 11. Computational calculated highest occupied molecular orbital (HOMO) and lowest unoccupied molecular orbital (LUMO) levels of 6FDI-2*o*BN. **a,** HOMO and LUMO distributions. **b,** Energy alignment of LUMO+1, LUMO, HOMO, and HOMO−1. The *Δ* values denote the energy level differences between the conformer #1 and each conformer. Note that 6FDI-2*o*BN possesses 35 conformers, thus, the three conformations were extracted to investigate the impact of molecular conformations on energy levels.

Supplementary Fig. 12. OLEDs with SOP-ETLs

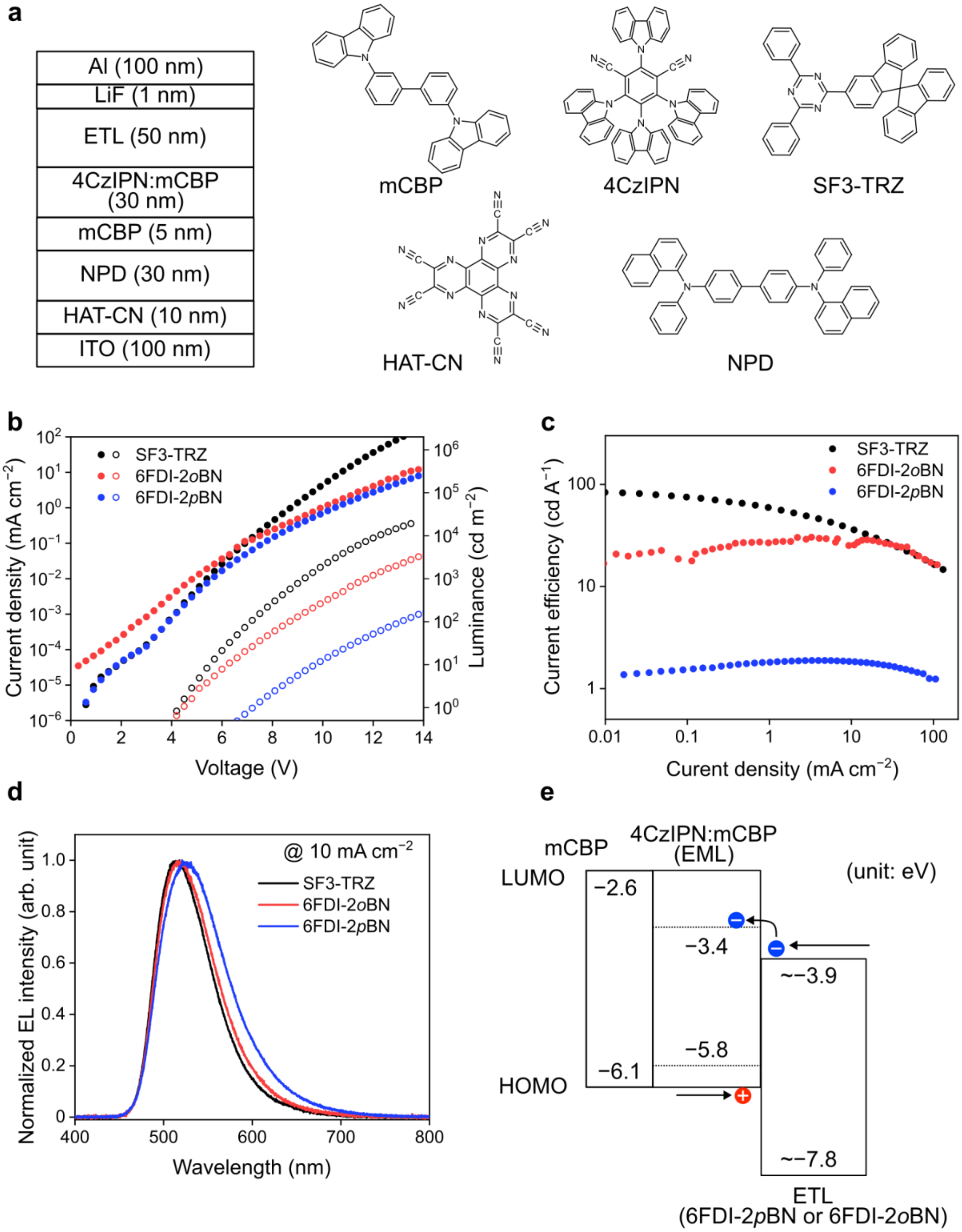

Supplementary Fig. 12. Device performance of organic light-emitting diodes (OLEDs) with SOP-ETLs. **a,** Device structure and molecular structures used in the device. **b,** Current density-voltage-luminance characteristics. **c,** Current efficiency (CE) profiles. **d,** Electroluminescence (EL) spectra at the current density of 10 mA cm^{-2}. **e,** Energy diagrams of OLEDs using electron transport layers (ETLs) with deep energy levels.

Supplementary Fig. 13. PL and EL lifetimes

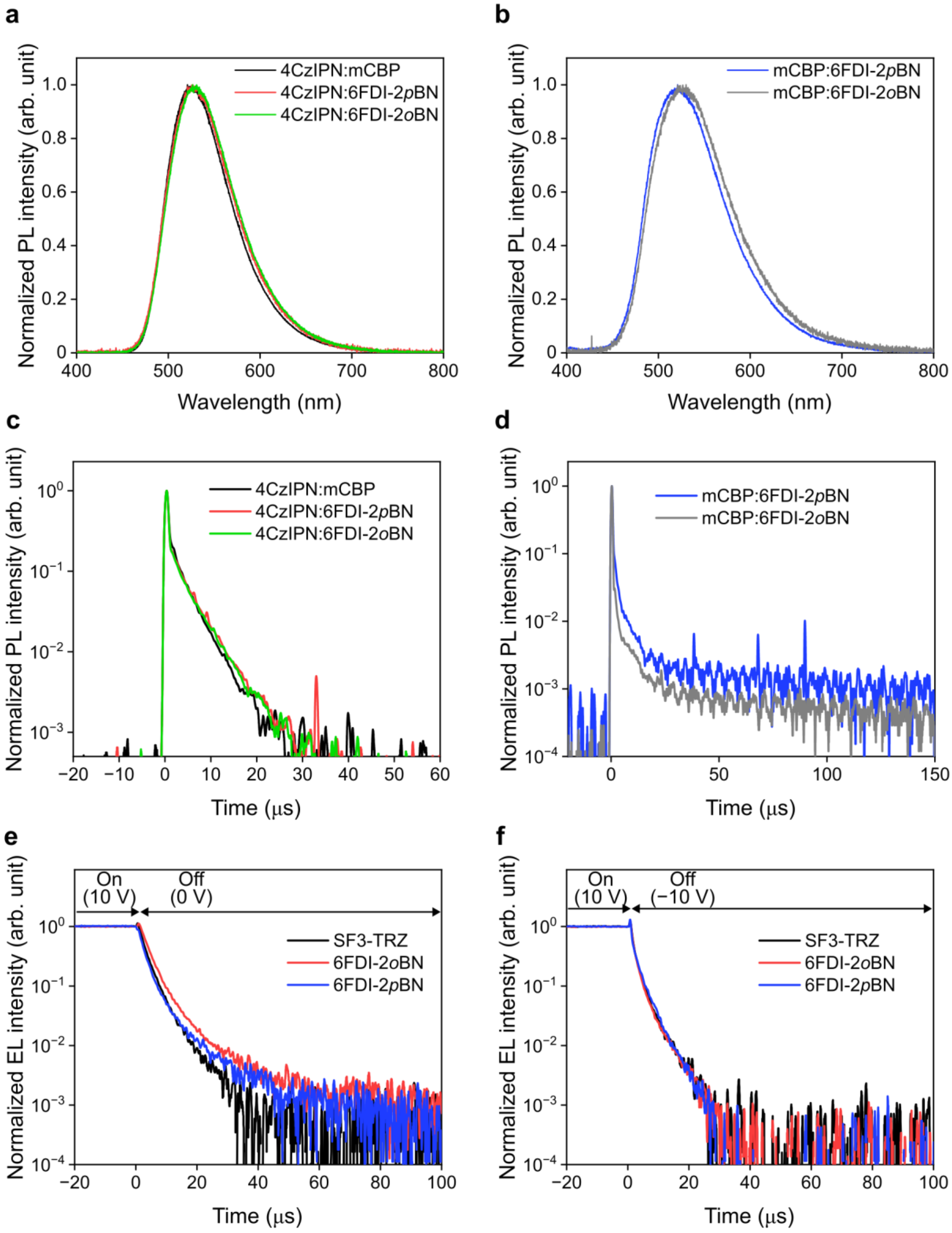

Supplementary Fig. 13. Photoluminescence (PL) of 4CzIPN in SOP layers and mCBP:ETL exciplex formation. **a,** PL spectra of 15 wt%-doped 4CzIPN in mCBP, 6FDI-2*p*BN, and 6FDI-2*o*BN. **b,** PL spectra of mixed films of mCBP and 6FDI molecules (50 wt%). **c,** Transient PL profiles of 15 wt%-doped 4CzIPN in mCBP, 6FDI-2*p*BN, and 6FDI-2*o*BN. **d,** Transient PL profiles of mixed films of mCBP and 6FDI molecules (50 wt%). **e,** Transient electroluminescence (EL) profiles of OLEDs with various ETL (applied voltage: 10 V). **f,** Transient EL profiles of OLEDs with various ETL (applied voltage: 10 V) with an off-bias of −10 V to remove the effect of stored charges on the transient profiles.

Supplementary Fig. 14. DCM profiles of OLEDs with SOP-ETLs

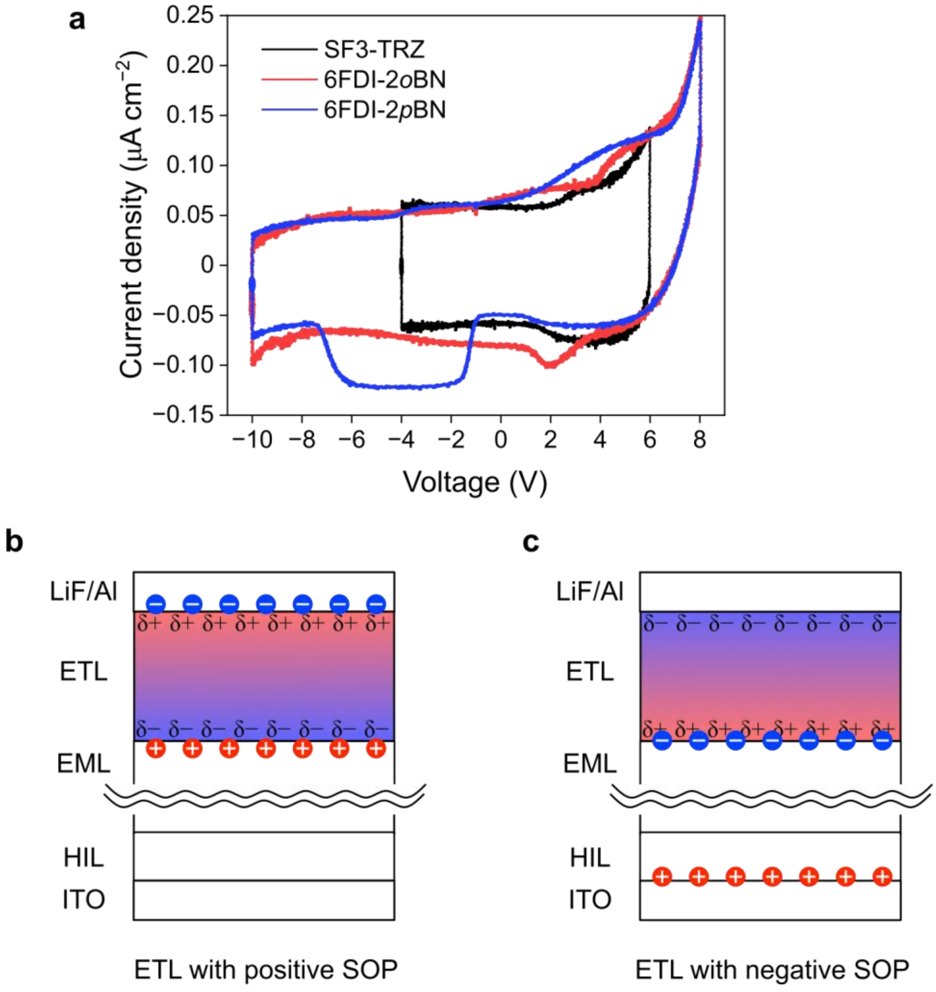

Supplementary Fig. 14. DCM results. **a,** Current density-voltage characteristics of OLEDs based on various ETLs measured using DCM. **b** and **c,** Schematic of estimated charge accumulation at the interfaces induced by polarization charges on the ETL interfaces with positive SOP (b) and negative SOP (c).

Supplementary Fig. 15. SOP-interlayers

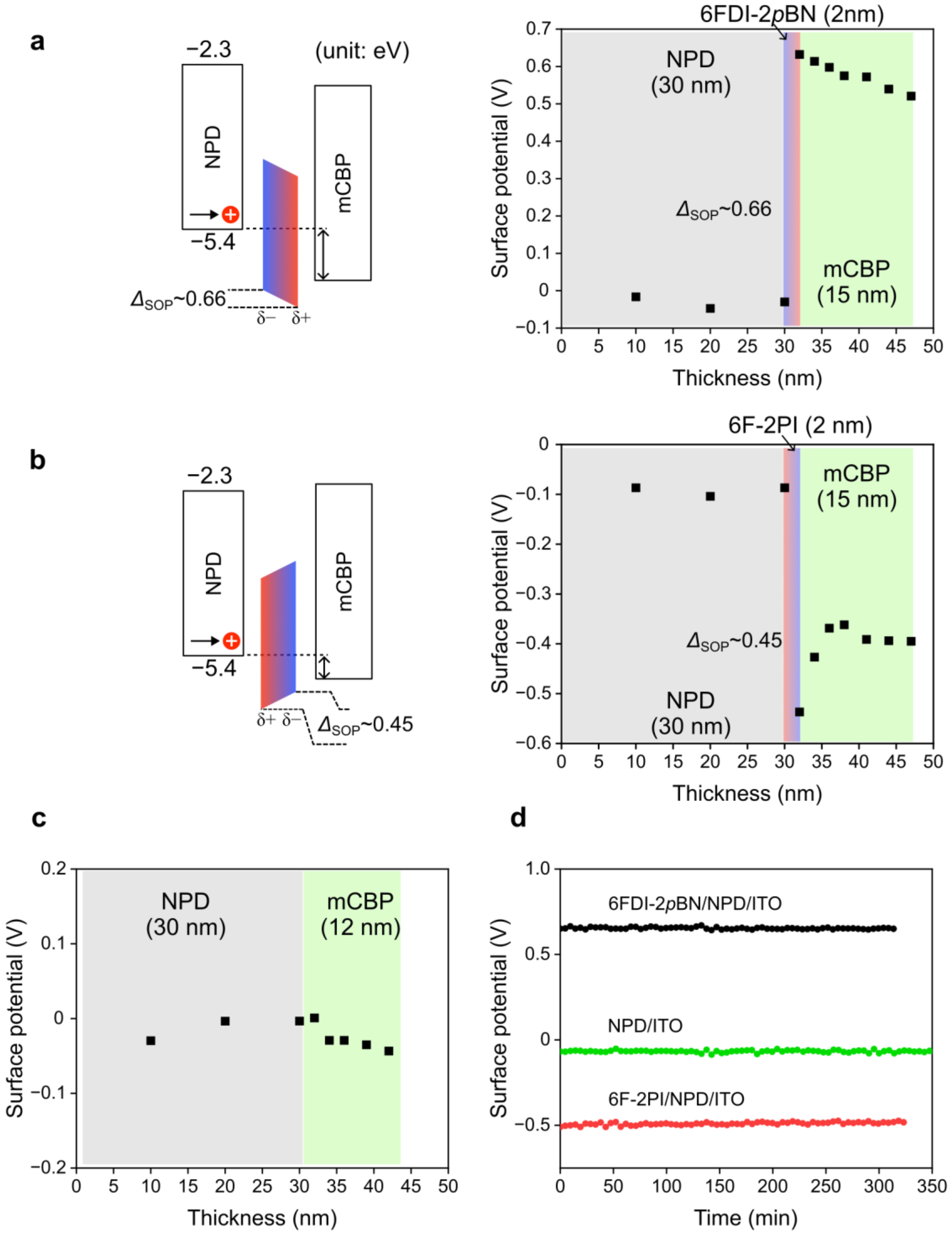

Supplementary Fig. 15. SOP interlayers. **a,** Energy level diagram of mCBP/6FDI-2*p*BN/NPD and surface potential profiles of mCBP (15 nm) / 6FDI-2*p*BN (2 nm) / NPD (30 nm) stack on an ITO substrate. The SOP-induced vacuum level shift (Δ_{SOP}) was determined using surface potential measurements. For the energy diagram, vacuum level shifts except for the Δ_{SOP} were ignored for simplicity. **b,** Energy level diagram of mCBP/6F-2PI/NPD and surface potential profiles of mCBP (15 nm) / 6F-2PI (2 nm) / NPD (30 nm) stack on an ITO substrate. **c,** Surface potential profiles of mCBP (12 nm) / NPD (30 nm) stack on an ITO substrate. **d,** Time dependence of surface potentials on NPD/ITO, 6FDI-2*p*BN/NPD/ITO, and 6F-2PI/NPD/ITO stacks under dark and vacuum condition.

Supplementary Fig. 16. DCM profiles of OLEDs with SOP-interlayers

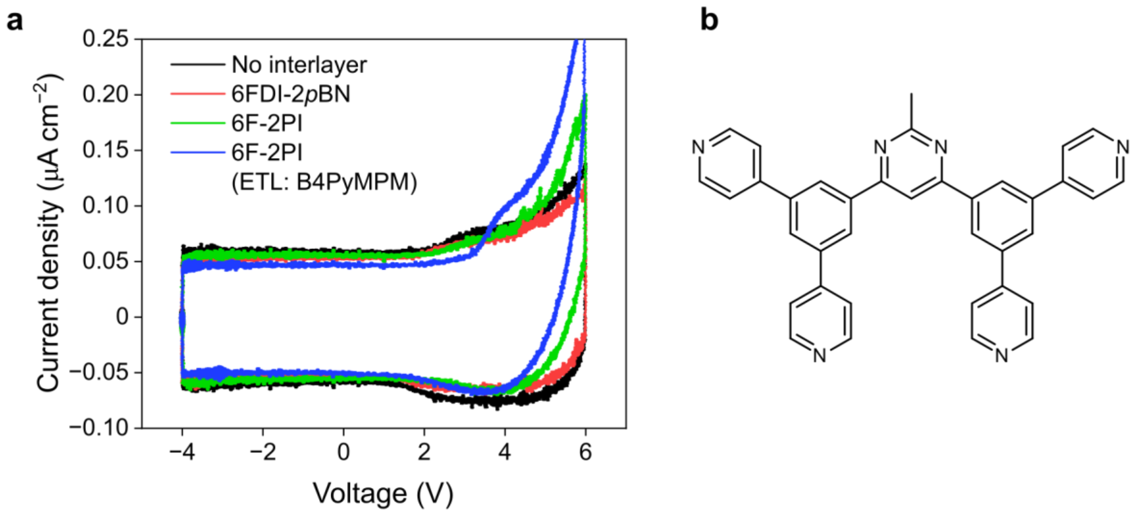

Supplementary Fig. 16. DCM profiles of OLEDs with SOP-interlayers. **a,** Current density-voltage characteristics of OLEDs based on various ETLs measured using DCM. **b,** Molecular structure of B4PyMPM.

Supplementary Table 5. OLED performance

Supplementary Table 5. OLED performance.

	V_{on} (V)[a]	V (V)[b]	CE_{max} (cd A^{-1})[c]	CE (cd A^{-1})[d]
**SOP ETL**				
SF3-TRZ (reference)	4.5	10.8	83.6	35.9
6FDI-2*p*BN	7.5	14.4	1.9	1.8
6FDI-2*o*BN	4.5	13.5	30.4	25.5
**SOP interlayer**				
No interlayer (reference)	4.5	10.8	83.6	35.9
6FDI-2*p*BN	6.9	13.2	56.4	28.5
6F-2PI	4.2	10.2	35.2	20.7
6F-2PI (B4PyMPM)	3.9	9.9	101.8	39.8

[a]Voltage at 1 cd m^{-2} (V_{on}). [b]Voltage at 10 mA cm^{-2}. [c]Maximum value of CE (CE_{max}). [d]CE value at 10 mA cm^{-2}.

Supplementary Figs. 17-30. NMR results

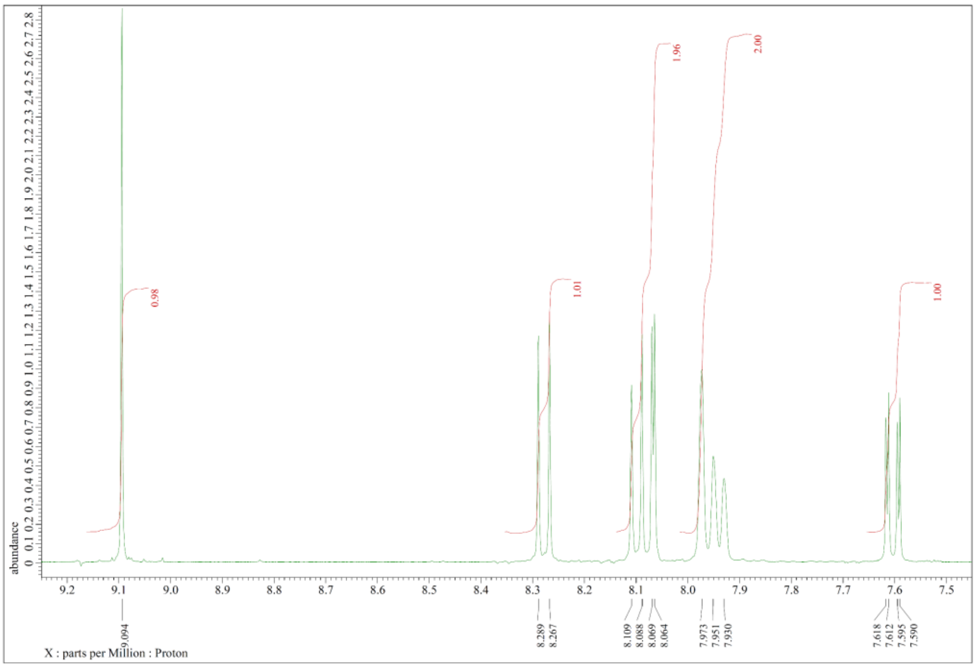

Supplementary Fig. 17. ^{1}H NMR spectrum of 6FDI-2BTA.

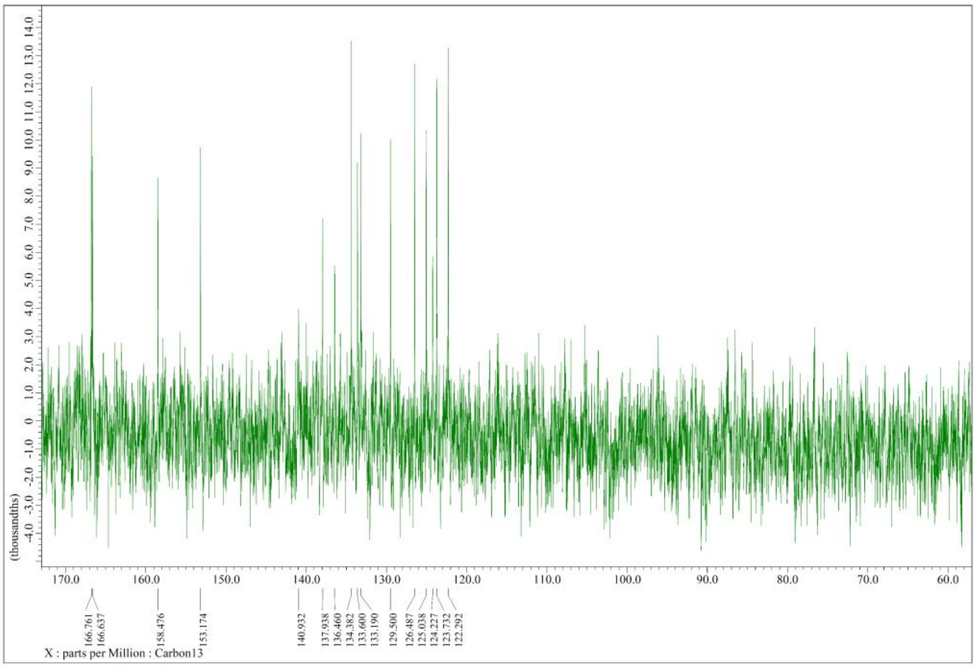

Supplementary Fig. 18. ^{13}C NMR spectrum of 6FDI-2BTA.

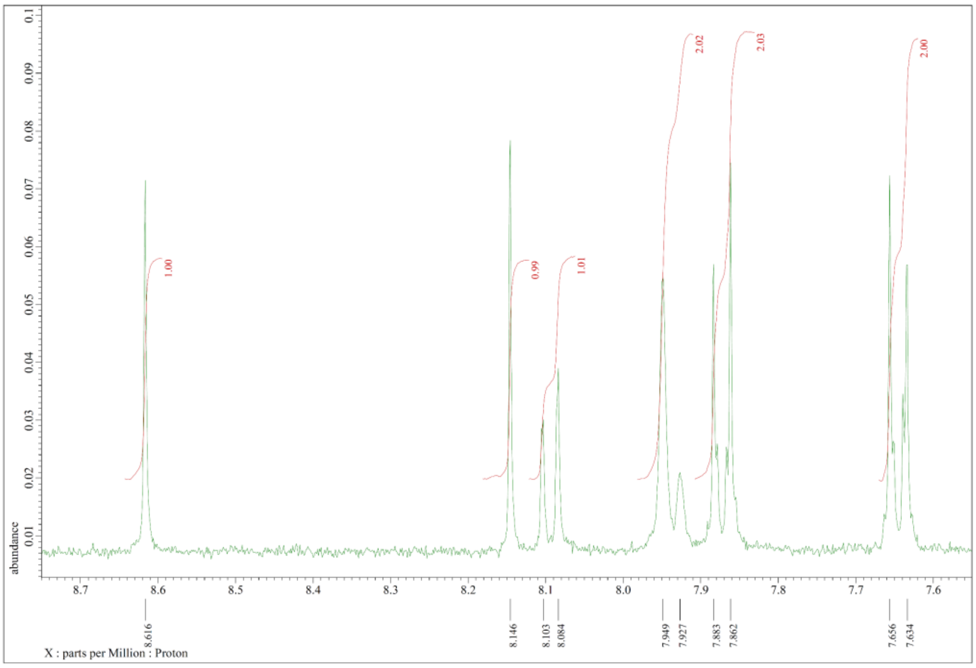

Supplementary Fig. 19. ^{1}H NMR spectrum of 6FDI-2TAZ.

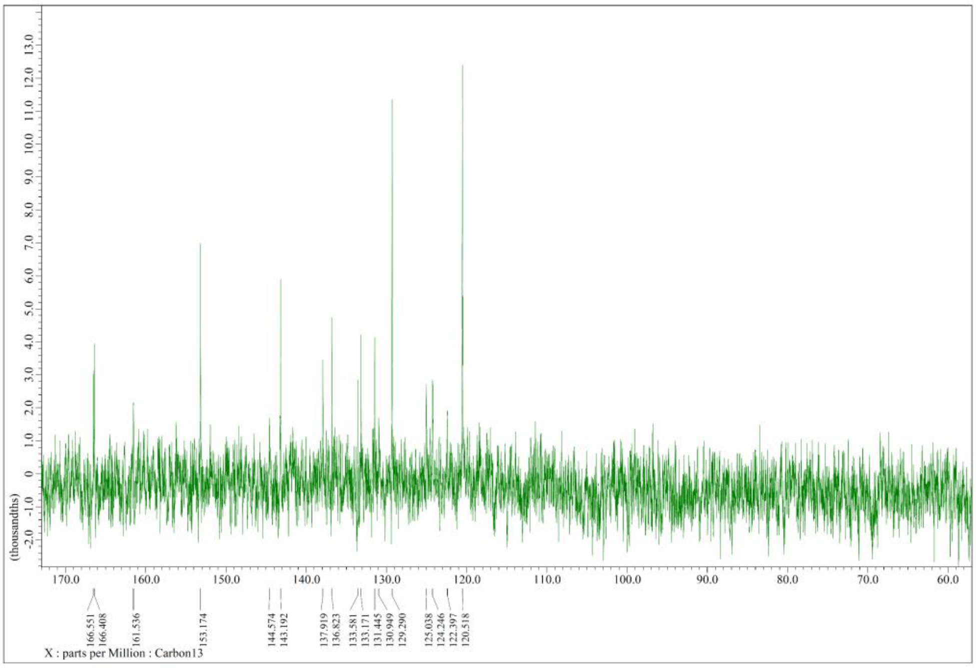

Supplementary Fig. 20. ^{13}C NMR spectrum of 6FDI-2TAZ.

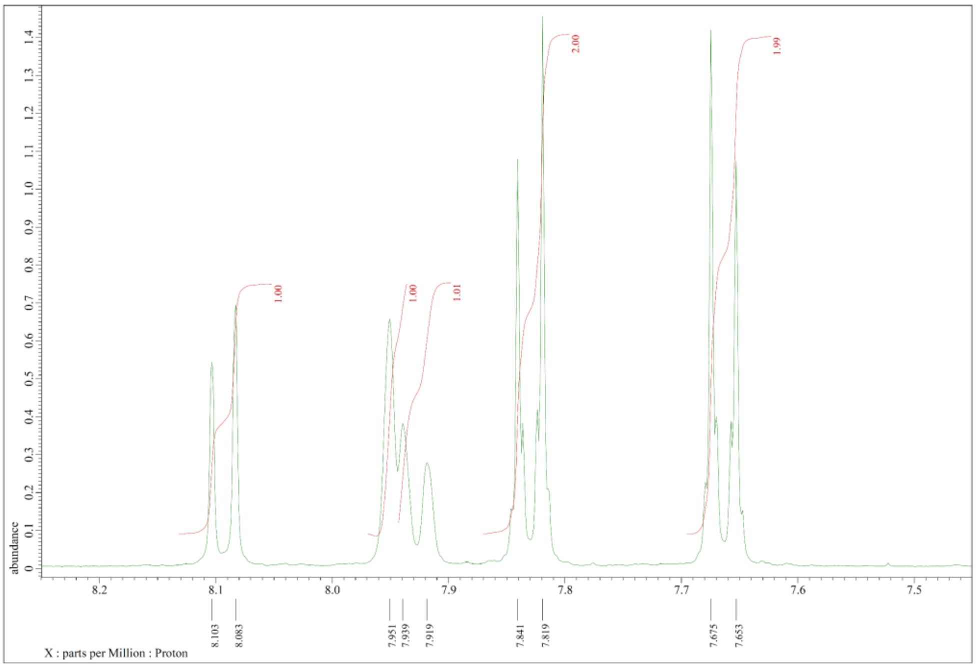

Supplementary Fig. 21. ^{1}H NMR spectrum of 6FDI-2*p*BN.

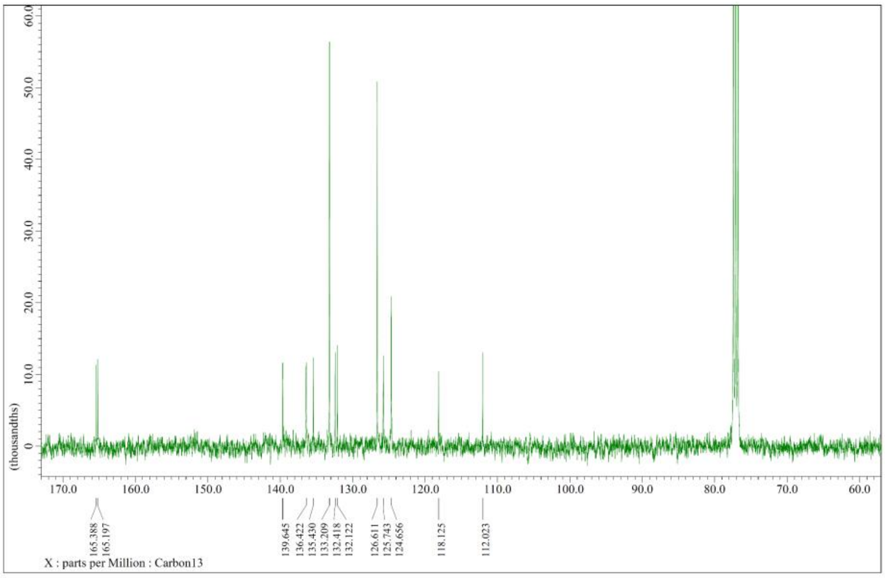

Supplementary Fig. 22. ^{13}C NMR spectrum of 6FDI-2*p*BN.

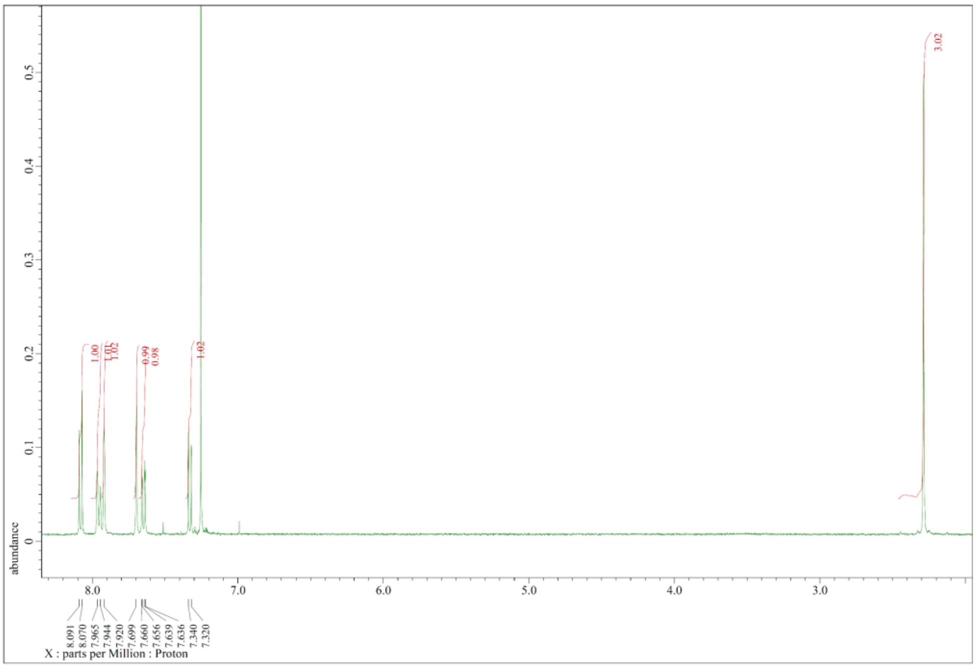

Supplementary Fig. 23. ^{1}H NMR spectrum of 6FDI-2*p*BNMe.

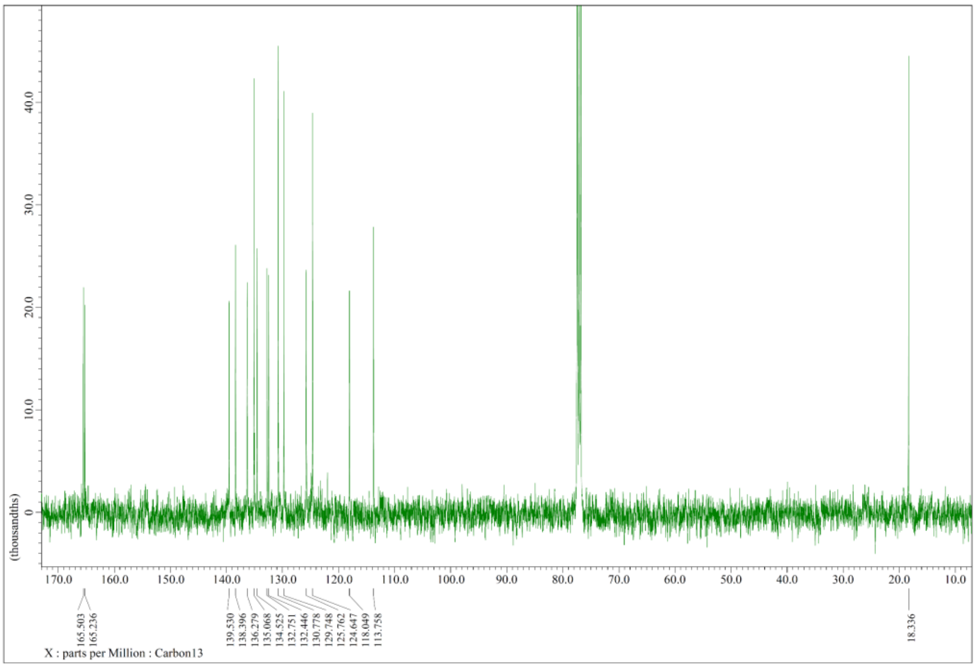

Supplementary Fig. 24. ^{13}C NMR spectrum of 6FDI-2*p*BNMe.

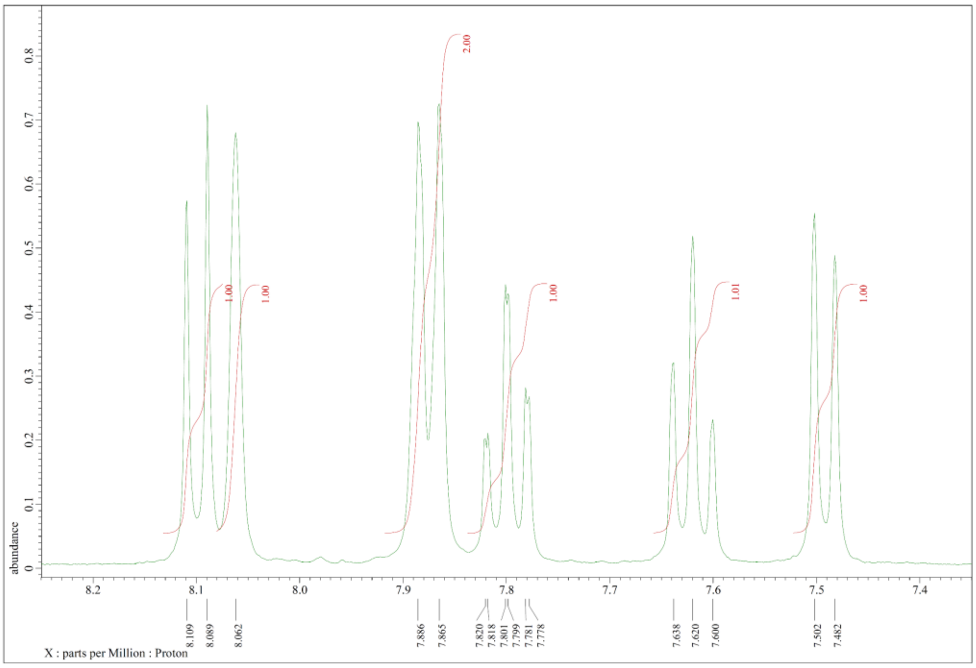

Supplementary Fig. 25. ^{1}H NMR spectrum of 6FDI-2*o*BN.

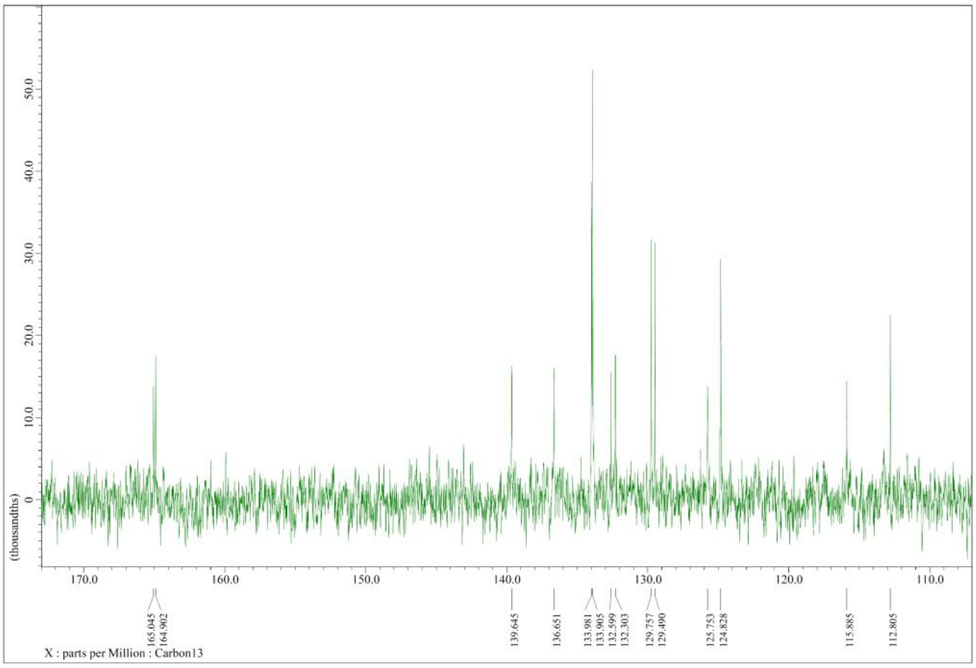

Supplementary Fig. 26. ^{13}C NMR spectrum of 6FDI-2*o*BN.

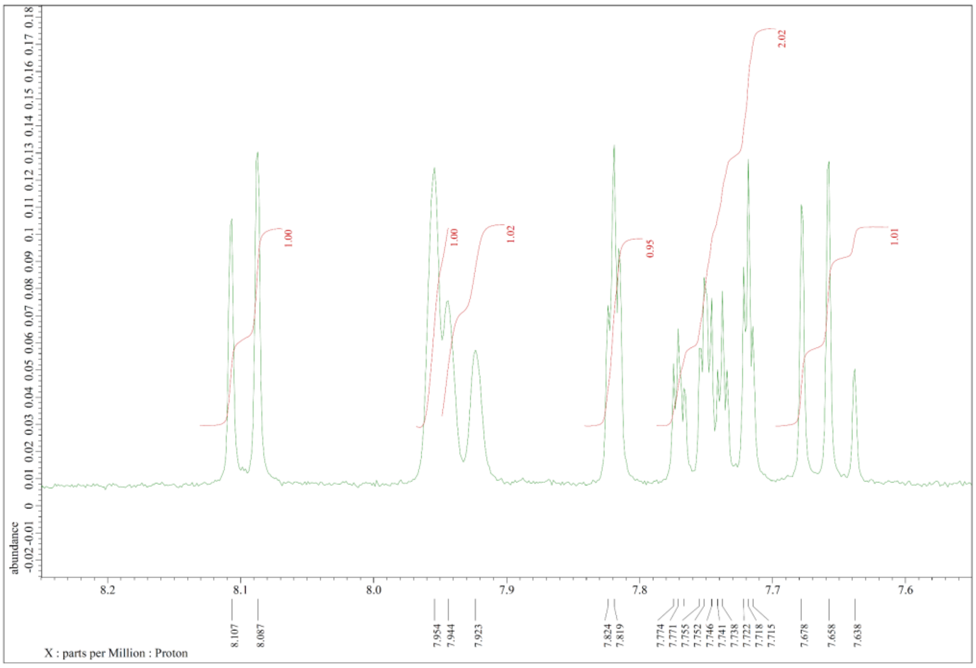

Supplementary Fig. 27. ^{1}H NMR spectrum of 6FDI-2*m*BN.

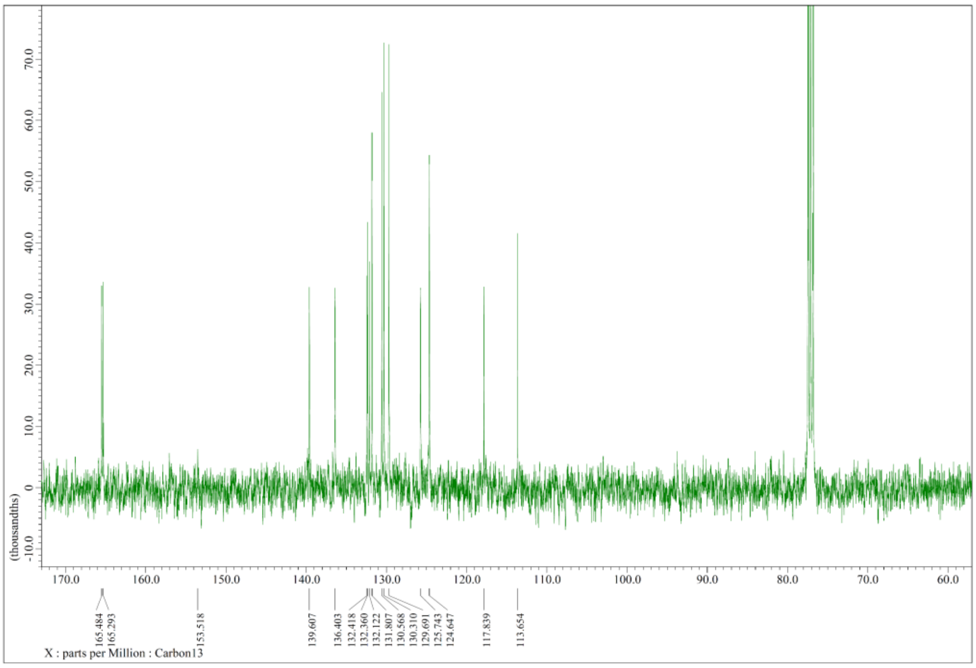

Supplementary Fig. 28. ^{13}C NMR spectrum of 6FDI-2*m*BN.

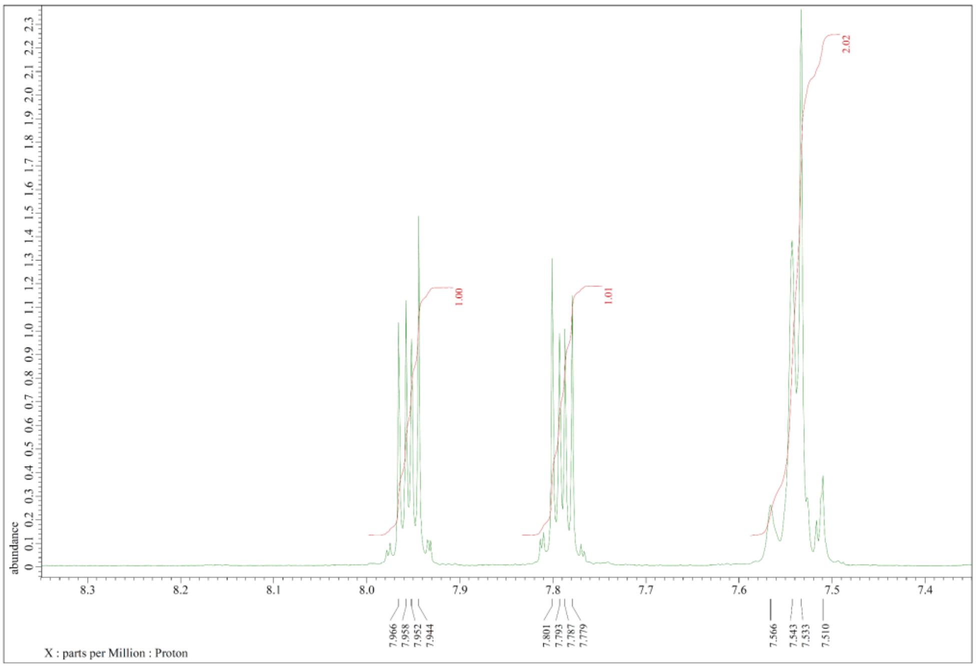

Supplementary Fig. 29. ^{1}H NMR spectrum of 6F-2PI.

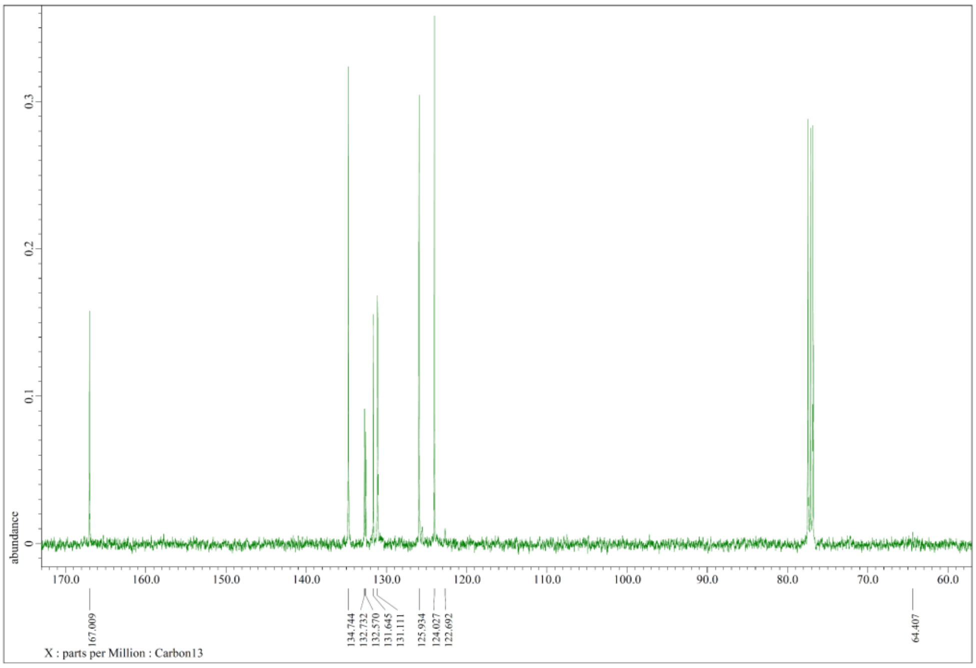

Supplementary Fig. 30. ^{13}C NMR spectrum of 6F-2PI.

Supplementary References

1. Noguchi, Y., Brütting, W. & Ishii, H. Spontaneous orientation polarization in organic light-emitting diodes. *Jpn. J. Appl. Phys.* **58**, SF0801 (2019).

2. Cakaj, A., Schmid, M., Hofmann, A. & Brütting, W. Controlling Spontaneous Orientation Polarization in Organic Semiconductors-The Case of Phosphine Oxides. *ACS Appl. Mater. Interfaces* **15**, 54721–54731 (2023).

3. Wang, W.-C., Nakano, K., Hashizume, D., Hsu, C.-S. & Tajima, K. Tuning Molecular Conformations to Enhance Spontaneous Orientation Polarization in Organic Thin Films. *ACS Appl. Mater. Interfaces* **14**, 18773–18781 (2022).

4. Tanaka, M., Auffray, M., Nakanotani, H. & Adachi, C. Spontaneous formation of metastable orientation with well-organized permanent dipole moment in organic glassy films. *Nat. Mater.* **21**, 819–825 (2022).
